# Combined oral immunization with probiotics Entercoccus faecalis delivering surface-anchored *Eimeria tenella* proteins provide protective efficacies against homologous infection in chickens

**DOI:** 10.3389/fimmu.2022.1042143

**Published:** 2022-10-13

**Authors:** Wenjing Zhi, Hang Chen, Bingrong Bai, Zhipeng Jia, Xinghui Pan, Biao Wang, Rui Kong, Qiuju Liu, Chunli Ma, Dexing Ma

**Affiliations:** ^1^ College of Veterinary Medicine, Northeast Agricultural University, Harbin, China; ^2^ Heilongjiang Provincial Key Laboratory of Pathogenic Mechanism for Animal Disease and Comparative Medicine, College of Veterinary Medicine, Northeast Agricultural University, Harbin, China; ^3^ College of Food Science, Northeast Agricultural University, Harbin, China

**Keywords:** immunization, *Entercoccus faecalis*, surface-anchored, *Eimeria tenella*, protective efficacy

## Abstract

**Background and Objectives:**

Avian coccidiosis is an intestinal parasitic disease exerting a highly negative impact on the global poultry industry. The aim of the present study is to evaluate the immune protective efficacies against *Eimeria tenella* infection in chickens orally immunized with combined recombinant probiotics *Entercoccus faecalis (E. faecalis)* delivering surface-anchored *E. tenella* proteins.

**Methods:**

Four kinds of novel probiotics vaccines that surface*-*expressing four *Eimeria tenella* (*E. tenella*) proteins EtAMA1, EtIMP1, EtMIC2 and Et3-1E were produced, respectively. The expression of four target proteins on the surface of recombinant bacteria was detected by Western blot and indirect immunofluorescence assay (IFA). Then the four kinds of recombinant *E. faecalis* were combined to immunize chickens *via* oral route in different combinations. The immunizations were performed three times at two-week intervals, and each for three consecutive days. After immunizations, chickens in each immunized group were orally challenged with *E. tenella* sporulated oocysts. The immune responses and protective efficacies against homologous infection were evaluated.

**Results:**

The results showed that three or four live recombinant *E. faecalis* induced effective antigen-specific humoral, intestinal mucosal immune responses, stimulated peripheral T lymphocytes proliferation, and displayed partial protections against homologous challenge as measured by cecal lesions, oocyst shedding, and body weight gain (BWG). Notably, higher levels of protective efficacies were observed when the four recombinant *E. faecalis* delivering target proteins were combined.

**Conclusion:**

Chickens orally administrated with three or four, especially the four combined recombinant *E. faecalis* stimulated specific immune responses, which provided anti-coccidial effects. This study offers an idea for future development of novel vaccines based on multi-antigens delivered by probiotic bacteria.

## Introduction

Avian coccidiosis, a kind of intestinal protozoan disease caused by the known seven bird *Eimeria* species, bring considerable economic burden to the poultry industry. Among the seven *Eimeria* species, *Eimeria tenella* (*E. tenella*) displays the strongest pathogenicity and leads to bloody diarrhea and severe cecal pathological lesions (1). Nowadays, conventional methods for preventing and controlling coccidiosis primarily rely on anti-coccidial drugs and live parasites vaccines. However, these common measures gradually display potential restrictions on poultry industry development with regard to drug-resistant *Eimeria* species, drug residues and food security (2). During *E. tenella* infection, many key proteins released from parasites play a vital role on invasion into host cells and eliciting immune responses (3). After invasion into cecal epithelial cells or glandular epithelium in submucosa, *E. tenella* parasites initiate and complete the multiple life cycle stages in chicken cecum (4). The in-depth study of key proteins that involved in invasion process and in immune responses is particularly important for exploring novel methods to prevent and control coccidiosis. Based on the published articles, four representative proteins secreted by *E. tenella* parasites interested us, including *E. tenella* apical membrane antigen 1 (EtAMA1), *E. tenella* microneme protein 2 (EtMIC2), *E. tenella* immune mapped protein-1 (EtIMP1) and *E. tenella* 3-1E protein. Previous study showed that there exist four steps during protozoan parasites invasion into host-cell, including attachment, apical reorientation, formation of moving junction and protective parasitophorous vacuole (PV) (5). It was reported that EtAMA1 was one of the various invasion-related proteins secreted by apical secretory organelles (6). Previous research demonstrated that a conserved ring-like structure between the surfaces of epithelial cells and invading protozoan parasites was formed, which is called moving junction and consist of rhoptry neck proteins (RONs) and apical membrane antigen 1 (AMA1) (7, 8). EtMIC2 is another important microneme protein and was recorded to be mainly localized in the anterior region and membrane of *E. tenella* sporozoites, in the cytoplasm of first and second-generation merozoites, and also in schizogony. Specific antibodies against EtMIC2 reduced the invasion of *E. tenella* sporozoites into host cells (9). Oral vaccination with *Saccharomyces cerevisiae* surface-displaying EtMIC2 protein contributed to the reduction of oocysts shedding and protective effects for birds against *E. tenella* infection (10). EtIMP1 is predicted to be a membrane protein of *E. tenella* and has been demonstrated to be immunogenic and confer protections against *E. tenella* challenge in birds. Previous reports evaluated the potential role of EtIMP1 as a vaccine candidate against *E. tenella* infections (11). Et3-1E protein is highly immunogenic and conserved among the seven *Eimeria* species, and is located on the outer surface of both sporozoites and merozoites in *E. tenella* life cycles. Several studies demonstrated recombinant 3-1E protein expressed in *Bacillus subtilis* WB600 (12), *Lactococcus lactis* NZ9000 (13), and *E. faecalis* (14) induced immune responses and provided partial protection against *E. tenella* infection. Considering the key roles of the above four proteins, we hypothesized that key proteins delivered by promising vector may induce host to produce specific and effective intestinal mucosal, humoral, and cell-mediated immune responses, which contribute to inhibition of parasites invasion and development in cecal epithelial cells.

As for delivery vehicles, several vectors were reported to deliver *Eimeria* proteins, such as bacteria (15–17), *Lactobacillus Plantarum* (18), nanoparticles (19), and genetically modified *Eimeria* parasites (20), and so on. Recently, genetically engineered lactic acid bacteria (LAB) was developed as new generation of vectors to deliver therapeutic proteins to mucosal tissue sites to evoke systemic and mucosal immunity (9, 21). Heterogenous proteins can be expressed in the cytoplasm, on the surface or secreted to the outside of LAB cells. Among the delivery tools of LAB, *E. faecalis* was recently reported as a promising vector to deliver target protein (14). Previously, we reported that Et3-1E protein, Hexon and Fiber 2 protein of fowl adenovirus 4 delivered by *E. faecalis* MDXEF-1, a kind of LAB isolated from ceca of healthy chickens and stored in our laboratory, induced protective immunity against homologous infection (14). However, whether combination of different recombinant live *E. faecalis* displaying surface-anchored key proteins that related to *Eimeria* infection provided more obvious immune protections against homologous infection remains unclear. In the present study, immune responses and protections against homologous challenge conferred by oral combined immunizations with four kinds of recombinant live *E. faecalis* expressing surface-anchored *E. tenella* antigens were evaluated and discussed.

## Materials and methods

### Animals, bacterial strains, and medium

One-day-old specific-pathogen-free (SPF) chickens were purchased from Harbin Veterinary Research Institute, Harbin, China. *Enterococcus faecalis* strain MDXEF-1 was stored in our lab in College of Veterinary Medicine, Northeast Agricultural University. *E. faecalis* MDXEF-1 was cultured in M17 medium (Qingdao Hope Bio-Technology Co., Ltd) with 0.5% glucose (GM17 medium) at 30 °C. The strains and plasmids used in this experiment were displayed in **Table 1**.

**Table 1:** Strains and plasmids used in this study.

Plasmids and bacteria	Relevant phenotype or genotype	Source or References
**Plasmids**		
pTX8048-SP-DC-Fiber2-CWA	modified plasmid used as template	Jia et al., 2021 (22)
pTX8048-SP-DC-EtAMA1-CWA	pTX8048 used to express EtAMA1 protein under control of nisin promoter	This study
pTX8048-SP-DC-EtIMP1-CWA	pTX8048 used to express EtIMP1 protein under control of nisin promoter	This study
pTX8048-SP-DC-EtMIC2-CWA	pTX8048 used to express EtMIC2 protein under control of nisin promoter	This study
pTX8048-SP-DC-Et3-1E-CWA	pTX8048 used to express Et3-1E protein under control of nisin promoter	This study
**Bacteria**		
MDXEF-1	*Enterococcus faecalis*, plasmid-free	Chen et al., 2020 (14)
MDXEF-1/EtAMA1	*E. faecalis*, EtAMA1-protein-producing probiotic strain	This study
MDXEF-1/EtIMP1	*E. faecalis*, EtIMP1-protein-producing probiotic strain	This study
MDXEF-1/EtMIC2	*E. faecalis*, EtMIC2-protein-producing probiotic strain	This study
MDXEF-1/Et3-1E	*E. faecalis*, Et3-1E-protein-producing probiotic strain	This study

### Construction of recombinant *E. faecalis* surface-anchoring *E. tenella* proteins

**Figure 1:**
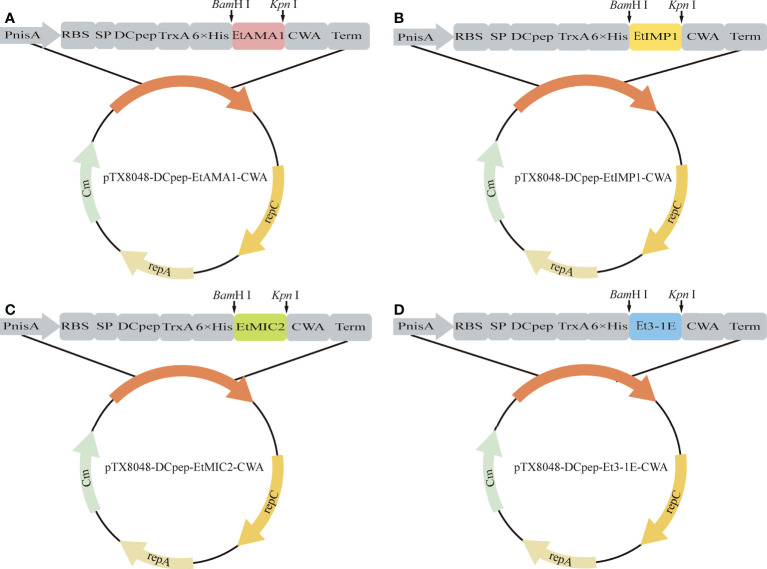
Schematic illustration of positive plasmids. The gene fragments EtAMA1, EtIMP1, EtMIC2 and Et3-1E digested by *Bam*H I and *Kpn* I were introduced into *Bam*H I/*Kpn* I sites of pTX8048-SP-DC-CWA to generate pTX8048-SP-DC-EtAMA1-CWA **(A)**, pTX8048-SP-DC-EtIMP1-CWA **(B)**, pTX8048-SP-DC-EtMIC2-CWA **(C)** and pTX8048-SP-DC-Et3-1E-CWA **(D)**, respectively.

The primers pair used for amplification of target fragment were designed according to the gene sequences of EtAMA1 (Accession NO. JN032081.1), EtIMP1 (Accession NO. KC215109.1), EtMIC2 (Accession NO. KC333870.1), and Et3-1E (Accession NO. EF426471.1) in NCBI GenBank. The target gene fragments EtAMA1, EtIMP1, EtMIC2, and Et3-1E were amplified according to the designed primers pair (**Table 2**), which were then digested with restriction enzyme *Bam*H I and *Kpn* I, respectively. To construct recombinant plasmids, the four digested fragments were subcloned into *Bam*H I/*Kpn* I site in plasmid pTX8048-SP-DC-Fiber2-CWA (22) that digested with the same two enzymes. The constructed plasmids pTX8048-SP-DC-EtAMA1-CWA, pTX8048-SP-DC-EtIMP1-CWA, pTX8048-SP-DC-EtMIC2-CWA, and pTX8048-SP-DC-Et3-1E-CWA were identified by sequencing and digestion with restriction enzyme, respectively. The target gene fragment was introduced to downstream of the nisA promoter in the four constructed plasmids, and the expressed proteins EtAMA1, EtIMP1, EtMIC2, and Et3-1E could be detected after induction by nisin (Sigma-Aldrich). The scheme for construction of target plasmids were shown in **Figure 1**. The four constructed plasmids were electrotransformed into *E. faecalis* MDXEF-1 strain, respectively. The selected bacteria colonies were cultured in GM17 medium and identified by enzyme digestion of plasmids that extracted from the cultured bacteria. The recombinant positive *E. faecalis* MDXEF-1/pTX8048-SP-DC-EtAMA1-CWA, MDXEF-1/pTX8048-SP-DC-EtIMP1-CWA, MDXEF-1/pTX8048-SP-DC-EtMIC2-CWA, and MDXEF-1/pTX8048-SP-DC-Et3-1E-CWA displaying surface-anchored target proteins were named MDXEF-1/EtAMA1, MDXEF-1/EtIMP1, MDXEF-1/EtMIC2, and MDXEF-1/Et3-1E, respectively.

**Table 2:** PCR primers pair used in the study.

Gene name	Accessin No.	Primers pair	Primer sequences (5′–3′)	Enzyme sites	Length of PCR products
EtAMA1	JN032081.1	EtAMA1-F	CGC*GGATCC*GTTCAACATAAATTACAACATAG	*Bam*H I	1269 bp
		EtAMA1-R	GG*GGTACC*GCCTCCTCCTTTACTTTCACA	*Kpn* I	
EtIMP1	KC215109.1	EtIMP1-F	CGC*GGATCC*ATGGGTGGTGCTTGCGGTAA	*Bam*H I	1191 bp
		EtIMP1-R	GG*GGTACC*TGTAGCTGCAACATTAC	*Kpn* I	
EtMIC2	KC333870.1	EtMIC2-F	CGC*GGATCC*ATGGCTAGAGCTTTATCATTAGTT	*Bam*H I	1041 bp
		EtMIC2-R	GG*GGTACC*TGATGATTGTTGTGTATCAGATTC	*Kpn* I	
Et3-1E	EF426471.1	Et3-1E-F	CGC*GGATCC*ATGGGTGAAGAAGCAGATAC	*Bam*H I	510 bp
		Et3-1E-R	GG*GGTACC*TTAAAATCCTCCTTGATAAAGAT	*Kpn* I	

### Preparation of cell wall protein samples

The selected colonies were cultured in 5 mL GM17 medium for 12 h, and 1.0 mL was inoculated in GM17 medium containing 10 μg/mL chloramphenicol. The expression of target protein was induced by adding nisin (Sigma-Aldrich) into the culture medium at OD600 value of 0.3, with a final concentration of 50 ng/mL. After cultivation for 5 h at 30 °C, the induced bacteria were collected by centrifugation at 4 °C by 10000 rpm for 2 min, washed twice with PBS (pH7.2). Then 1.0 mg/mL lysozyme was used to digest cell wall to prepare protein samples according to previously reported method (23).

### Western blot

The target protein was separated by sodium dodecyl sulfate-polyacrylamide gel electrophoresis (SDS-PAGE) and then detected by Western blot as described in previous report (24). Briefly, the protein samples were separated by 10% SDS-PAGE and transferred to nitrocellulose membrane. The membrane was blocked by TBST buffer (0.01% Tween-20, Tris-basel 20 mmol/L pH 7.5, NaCl 100 mmol/L) containing 5% skimmed milk at 37 °C for 2 h, and then incubated with primary antibodies at 4 °C overnight, including rabbit anti-Et3-1E antibody (24), rabbit anti-EtAMA1 antibody (25), rabbit anti-EtIMP1 antibody (26) and rabbit anti-His tag antibody (Sigma, Aldrich) with dilution of 1:1000 for all the four primary antibodies. Each membrane was washed four times with TBST buffer. Then the membrane was reacted with goat-anti rabbit (ZSGB-Bio, Beijing, China) diluted by 1:2500 for 1 h at room temperature and washed four times with TBST buffer. The protein was observed under imaging system (ChemiScope series 3100).

### Indirect immunofluorescence

The four recombinant *E. faecalis* induced by 50 ng/mL nisin were collected and washed three times with PBS (pH7.2), incubated with the corresponding primary antibodies (1:500) at 37 °C for 2 h, and then washed three times with PBS (pH7.2). Fluorescein isothiocyanate (FITC)-labeled goat anti-rabbit IgG was used as secondary antibody (1:200) to react with the bacteria in dark at 37 °C for 2 h. The bacteria were washed three times and then observed under the fluorescence microscope (Leica DM2000).

### Vaccinations and challenge experiment

**Figure 2:**
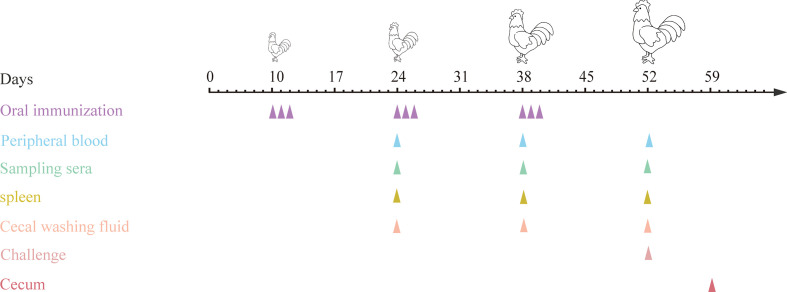
Procedures for immunizations, challenging and tissues sampling during animal experiments. At 10, 24 and 38 days of age, chickens were orally immunized with live *E. faecalis* expressing surface-anchored protein in different combinations. Immunizations were conducted for three times at intervals two weeks, and each for three consecutive days and once a day. On day 14 after the third immunization, chickens except in PBS group were challenged with sporulated *E. tenella* oocysts. At two weeks after each immunization, peripheral blood were sampled for preparing sera and T lymphocytes, spleens were collected for detection of cytokines, and ceca were sampled for preparing cecal lavage fluid. On day 7 post challenging, ceca were sampled for quantification of inflammatory cytokines.

10-day-old specific pathogen free (SPF) chickens were randomly divided into eight groups (20 chickens in each group) (**Table 3**). The first immunization was performed at 10 days of age, and all chickens were weighed before vaccination. *E. tenella* infected control group and uninfected control group (PBS group) were designed. Each chicken in the two control groups was orally gavaged with 200 μL PBS (pH7.2), and group 3 was orally gavaged with 4.0 × 10^10^ CFU (200 μL) MDXF-1/pTX8048 (vector control). Groups 4, 5, 6, 7 and 8 were designed as combined vaccination groups, sequentially including three combined live bacteria MDXEF-1/EtAMA1/EtIMP1/EtMIC2 (group 4), MDXEF-1/EtAMA1/EtIMP1/Et3-1E (group 5), MDXEF-1/EtAMA1/EtMIC2/Et3-1E (group 6) and MDXEF-1/EtIMP1/EtMIC2/Et3-1E (group7), and four combined live recombinant bacteria MDXEF-1/EtAMA1/EtIMP1/EtMIC2/Et3-1E (group 8), respectively. Chickens in groups 4, 5, 6, 7 and 8 were orally gavaged with corresponding three or four kinds of live recombinant *E. faecalis*, and each chicken with 1.0× 10^10^ CFU (50 μL) of each recombinant live bacteria. In addition, 50 μL (1.0× 10^10^ CFU) of MDXF-1/pTX8048 was meanwhile orally gavaged in the groups 4, 5, 6 and 7, respectively, to maintain the total immunization volume of 200 μL per chicken. All chickens were received the second and the third immunization at 24 and 38 days of age, respectively. Each immunization was conducted once a day and for three consecutive days at intervals two weeks. On day 14 post the third immunizations, chickens from all groups except the PBS group were challenged with 2.0×10^4^ freshly harvested *E. tenella* sporulated oocysts. The immune procedures are shown in **Figure 2**. Anticoccidial-free feed and water were provided ad libitum during the whole trial. Animal experiments were performed according to the regulations of the Ethics Committee for Animal Sciences at Northeast Agricultural University, Heilongjiang Province, China (NEAUEC20210332).

**Table 3:** Experimental design for animal experiments.

Groups	number of chickens	Immunizations with Live recombinant E. faecalis, total 4.0×10^9^ CFU (200 μL) per chicken					
		PBS (pH7.2)	MDXF-1/pTX8048	MDXEF-1/EtAMA1	MDXEF-1/EtIMP1	MDXEF-1/EtMIC2	MDXEF-1/Et3-1E
**PBS control group**	20	○	**/**	**/**	**/**	**/**	**/**
***E.tenella* control group**	20	○	**/**	**/**	**/**	**/**	**/**
**MDXEF-1/pTX8048(Vector control group)**	20	**/**	●	**/**	**/**	**/**	**/**
**MDXEF-1/EtAMA1/EtIMP1//EtMIC2**	20	**/**	◆	◆	◆	◆	**/**
**MDXEF-1/EtAMA1/EtIMP1/Et3-1E**	20	**/**	◆	◆	◆	**/**	◆
**MDXEF-1/EtAMA1/EtMIC2/Et3-1E**	20	**/**	◆	◆	**/**	◆	◆
**MDXEF-1/EtIMP1/EtMIC2/Et3-1E**	20	**/**	◆	**/**	◆	◆	◆
**MDXEF-1/EtAMA1/EtIMP1/EtMIC2/Et3-1E**	20	**/**	**/**	**◇**	**◇**	**◇**	**◇**

The four recombinant E. faecalis delivering four surface-anchored proteins EtAMA1, EtIMP1, EtMIC2 and Et3-1E were named MDXEF-1/EtAMA1, MDXEF-1/EtIMP1, MDXEF-1/EtMIC2, MDXEF-1/Et3-1E, respectively.

○ each chicken in PBS group was orally gavaged with 200 μL PBS (pH7.2).

● each chicken in vector control group was orally vaccinated with 4.0 ×10^9^ CFU (200 μL) live recombinant E. faecalis MDXEF-1/PTX8048.

◆ each chicken was orally vaccinated with 1.0 ×10^9^ CFU (50 μL) of live recombinant E. faecalis MDXEF-1/pTX8048, MDXEF-1/EtAMA1, MDXEF-1/EtIMP1, MDXEF-1/EtMIC2 and MDXEF-1/Et3-1E, respectively.

◇each chicken was orally immunized with 1.0 ×10^9^ CFU (50 μL) of live recombinant E. faecalis MDXEF-1/EtAMA1, MDXEF-1/EtIMP1, MDXEF-1/EtMIC2 and MDXEF-1/Et3-1E, respectively.

### Peripheral blood T lymphocyte proliferation

On day 14 post each oral vaccination, proliferation of chicken peripheral blood T lymphocytes was detected with CCK-8 kit (G021-1-2, Nanjing Jiancheng, Nanjing, China) with some modifications. Briefly, 3.0 mL of peripheral blood was collected from each chicken by using anticoagulant blood tube *via* the wing vein, then T lymphocytes from chickens in each group (n=3) were isolated by using lymphocyte isolation medium (1.005 g/ml) (Tianjin Haoyang, China). 100 μL of lymphocyte suspension (5.0×10^5^ cells/mL) was added to each well. Then 100 μL of concanavalin A (ConA) or mixed four target proteins was added to each well with a final concentration of 20 μg/mL and 20 μg/mL (5μg/mL for each protein), respectively. After cultivation for 48 h at 37 °C, 10 μL of CCK-8 solution was added to each well to culture for another 4 h. The OD450 nm values in each well were detected using ELISA reader (Bio-Rad, USA).

### Mucosal sIgA and serum IgG levels

The levels of antigen-specific IgG in sera and secretory IgA (sIgA) at mucosal surface in ceca were tested by using indirect enzyme-linked immunosorbent assay (ELISA). At two weeks after each immunization, chickens (n=3) were randomly selected from each group for collecting peripheral blood *via* wing vein. The collected blood were incubated at 37°C for 30 min, followed by refrigeration at 4°C for 2 h, and then centrifuged at 3500 rpm at 4°C. The supernatant was collected for preparing serum used for detection of sera IgG that specifically induced by target antigens. The above selected three chickens from each group were then euthanized, and 5 centimeters of ceca were sampled. 1.5 ml of PBS (containing 2 μg/mL protease inhibitors) was used to wash cecal mucosal surface three times. The cecal lavage fluid were collected and centrifuged at 3500 rpm at 4°C for 10 min, and the supernatants were harvested for detecting levels of sIgA induced by target antigens. The plates were coated with 100 μL (10 μg/mL) of recombinant *E. tenella* protein EtAMA1, EtIMP1, Et3-1E and EtMIC2 expressed in *E. coli* BL21, respectively, then blocked with 5% skimmed milk at 37 °C for 2 h. After washing three times with PBST buffer (PBS with 0.05% Tween-20), the prepared sera (1:100 dilution) and cecal lavage fluids (1:50 dilution) were added into wells in the plates and incubated for 2 h at 37 °C. After washing three times with PBST, HRP-conjugated goat anti-chicken IgG (1:5000) or IgA (1:5000) was added as secondary antibody to react at 37 °C for 1 h. 100 μL of 3, 3’, 5, 5’-tetramethylbenzidine (TMB) chromogenic solution (Solarbio, China) was added to each well to stop reaction. The OD values at 450 nm in each well was measured by enzyme labeling instrument (Bio-Rad, USA).

### Quantification of cytokines by qRT-PCR post three immunizations

At two weeks post each immunization, chickens (n=3) were randomly chosen from each group and euthanized. The spleens were sampled, snap-frozen in liquid nitrogen, and then stored at -80°C until further analysis. The spleens were homogenized with tissue homogenizer (Bioprep-24, China). Total RNA was extracted from tissue homogenates using the GenElute Mammalian RNA Miniprep Kit (83913-1EA, Sigma-Aldrich, China) according to the provided protocol. Then cDNA was prepared according to the method described by Wang et al. (27).The levels of several cytokines including chicken interleukin 2 (IL-2) (Accession NO. NM_204153.2), IL-4 (Accession NO. NM_001007079.2), IL-10 (Accession NO. NM_001004414.4), IL-15 (Accession NO. NM_204571.2), and chicken interferon (IFN-γ) (Accession NO. NM_205149.2) were quantified by real-time PCR (qRT-PCR). β-actin (Accession NO. NM_205518.2) was selected as endogenous reference gene. The primers used for quantification were listed in **Table 4**. 2 ^−ΔΔCT^ method (28) is used to standardize data.

**Table 4:** Primer sequences for qRT-PCR quantification.

Gene name	Accessin No.	Primers pair	Primer sequences (5′–3′)	Length of PCR products
β-actin	NM_205518.2	β-actin/F	GCCAACAGAGAGAAGATGACAC	140 bp
		β-actin/R	GTAACACCATCACCAGAGTCCA	
IL-2	NM_204153.2	IL-2/F	GTGGCTAACTAATCTGCTGTCC	105 bp
		IL-2/R	GTAGGGCTTACAGAAAGGATCAA	
IL-4	NM_001007079.2	IL-4/F	CTGTGCCCACGCTGTGCTTA	83 bp
		IL-4/R	GGAAACCTCTCCCTGGATGTCA	
IL-10	NM_001004414.4	IL-10/F	GGCTCACTTCCTCCTCC	94 bp
		IL-10/R	TGACTTTCACCTGCAGATG	
IL-15	NM_204571.2	IL-15/F	TGCCAGGAACCTGTAATGAGATGT	100 bp
		IL-15/R	TGTTCCGTACATCATGCTTCCTAC	
IFN-γ	NM_205149.2	IFN-γ/F	CAAAGCCGCACATCAAACA	80 bp
		IFN-γ/R	TTTCACCTTCACGCCATC	

### Quantification of intestinal inflammatory cytokines by ELISA post challenge

On day 7 post *E. tenella* infection, three chickens were selected from each group and euthanized. Ceca samples were sampled and store at -80°C for quantifying protein levels of proinflammatory cytokines. 100 mg of ceca tissues were homogenized by using tissue homogenizer (Bioprep-24, China). The protein levels of IL-6, IL-8, IL-17, and interleukin 1 beta (IL-1β) were quantified using ELISA kit (ml059839, ml059840, ml059833, and ml059835, Shanghai enzyme-linked, Shanghai, China) according to the manufacturer’s protocol.

### Pathological changes in ceca

On day 7 post challenge by *E. tenella*, cecal samples from chickens randomly selected from each group were collected, and gross pathological changes in ceca were recorded. Meanwhile, five millimeters caca were fixed in freshly prepared 10% neutral buffered formalin. The fixed tissue samples were used to prepare histopathological slides according to common steps including embedding by paraffin, section at 5 μm thickness, and staining with hematoxylin and eosin (HE). The prepared slides were observed by three pathologists independently under a light microscope (Nikon, EX200) to record the histopathological lesions in ceca.

### Immune protective efficacy

Five chickens were chosen from each group to assess cecal lesions based on scoring method as previously described by Johnson and Reid (29). Chickens from all groups were weighed at 10 (before first vaccination), 24 (before secondary vaccination), 38 (before third vaccination), 52 (before challenging), and 59 days of age (at 7 days post challenge). Body weight gains (BWG) were calculated as follows: BWG = body weight at 7 days post challenge (59-day-old)−body weight when challenging (52-day-old). For oocyst counting, feces from each chicken within one group that was raised in ten cages were harvested, respectively, between days 5 and 7 post challenge. Oocysts per gram of feces were counted under the microscope according to the reported method (26).

### Statistical analysis

All the data were analyzed using SPSS 26.0 software (SPSS, Chicago, IL, USA). All statistical analyses were performed by the way of one-way ANOVA and Duncan’s multiple-comparison procedures. A p-value of less than 0.05 is considered to be significant. All results were expressed as mean ± standard deviation (SD). The GraphPad Prism 9.0 (GraphPad Software, USA) was used for graphical processing.

## Results

### Construction and expression of target proteins in recombinant E. faecalis

**Figure 3:**
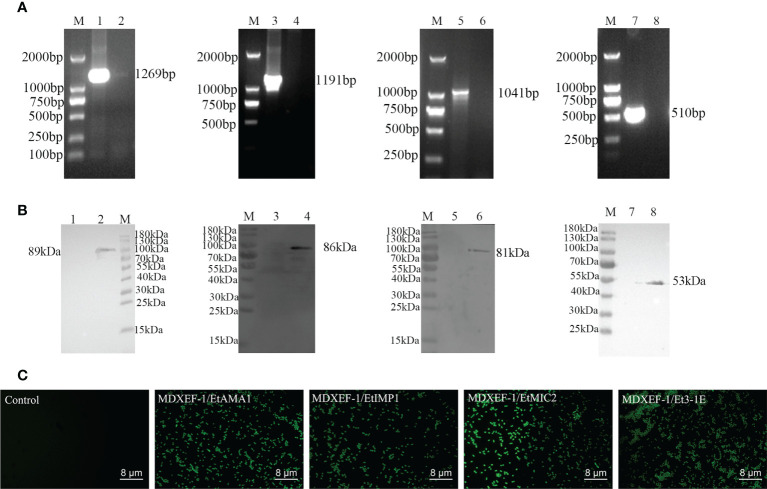
Identification of positive plasmids and recombinant *E. faecalis* expressing surface-anchored target proteins. **(A)** Identification of positive bacteria by colony PCR. Lane M, DNA Marker (DL 2000); Lane 1: Amplified EtAMA1 fragment. Lane 3: Amplified EtIMP1 fragment Lane 5: Amplified EtMIC2 fragment. Lane 7: Amplified Et3-1E fragment. Lane 2, 4, 6 and 8: Negative control. **(B)** Western blot analysis of expressed proteins in recombinant *E. faecalis*. M: Molecular weight marker. Lane 2, 4, 6 and 8: EtAMA1, EtIMP1, EtMIC2 and Et3-1E protein expressed in MDXEF/EtAMA1, MDXEF/EtIMP1, MDXEF/EtMIC2 and MDXEF/Et3-1E, respectively. Lane 1, 3, 5 and 7: Negative control MDXEF/pTX8048. **(C)** Detection of surface-displayed proteins using indirect immunofluorescence assay (IFA).

All the four positive plasmids pTX8048-SP-DC-EtAMA1-CWA, pTX8048-SP-DC-EtIMP1-CWA, pTX8048-SP-DC-EtMIC2-CWA, and pTX8048-SP-DC-Et3-1E-CWA were characterized and sequenced, which were then transformed into *E. faecalis* MDXEF-1 by electroporation, respectively. The four recombinant live *E. faecalis* were identified by colony PCR and double-enzyme digestion of target plasmids that extracted from positive bacteria **(** **Figure 3A**). The four recombinant *E. faecalis* delivering surface-anchored proteins EtAMA1, EtIMP1, EtMIC2 and Et3-1E were named MDXEF-1/EtAMA1, MDXEF-1/EtIMP1, MDXEF-1/EtMIC2, MDXEF-1/Et3-1E, respectively. Western blot was applied to detect target protein bands expressed in the four recombinant *E. faecalis*, showing expected band of 89 kDa, 86 kDa, 81 kDa and 53 kDa, respectively (**Figure 3B**). To further confirm these results, the expression of four target proteins was detected with indirect fluorescence assay (IFA). It was discovered that bacteria delivering target protein displayed noticeable fluorescence, while bacteria with empty vector showed no fluorescence (**Figure 3C**), demonstrating that four target proteins EtAMA1, EtIMP1, EtMIC2 and Et3-1E were expressed on the surface of recombinant bacteria, respectively.

### Peripheral T lymphocytes proliferation

**Figure 4:**
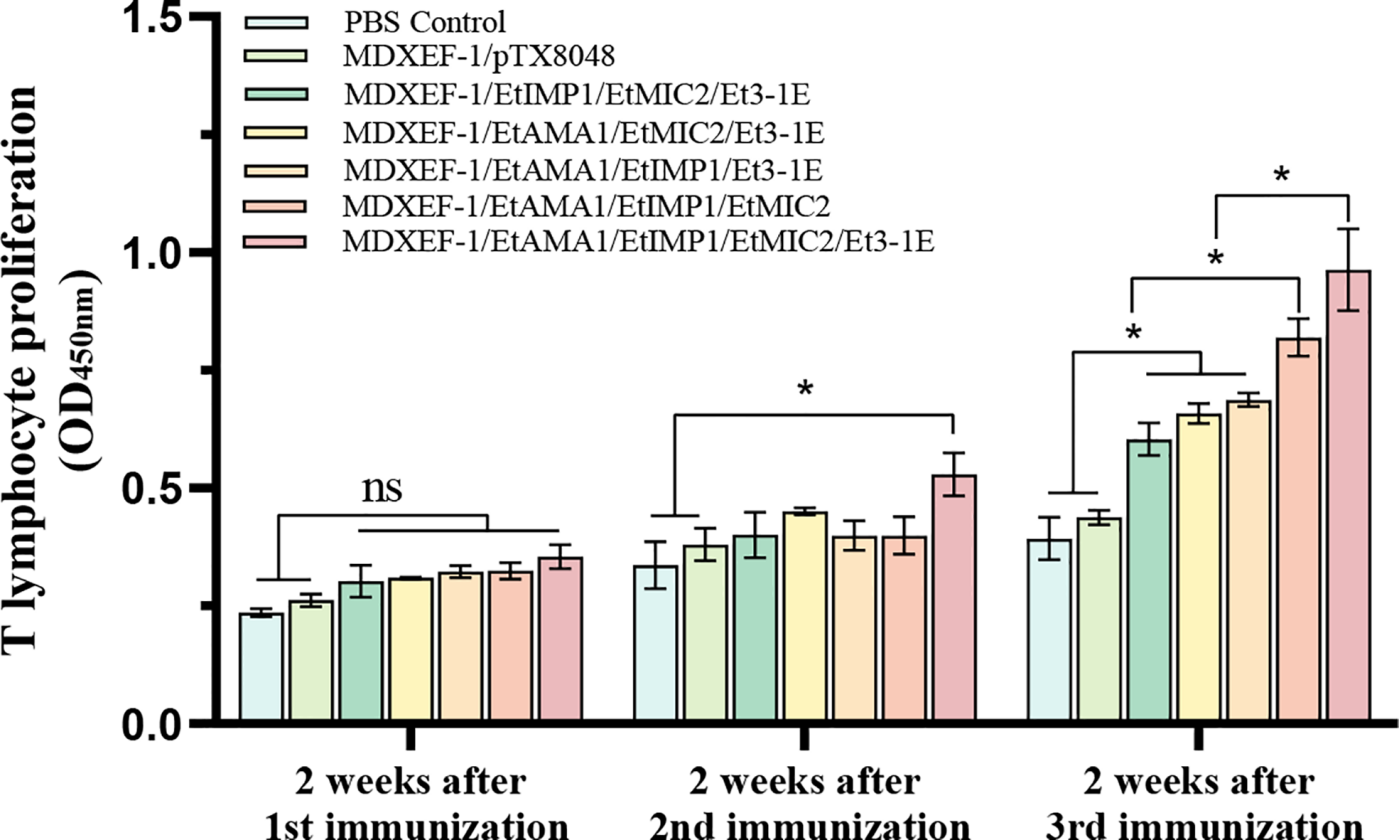
Proliferation of T lymphocytes in peripheral blood of chickens. At 2 weeks after each vaccination, the isolated peripheral blood T lymphocytes were cultured in 96-well plate (5.0 × 10^4^ cells per well), and then were stimulated with concanavalin A (ConA) (20 μg/ml) or mixed four target proteins (5 μg/ml for each protein) expressed in *E. coli* BL21. CCK-8 kit was utilized to detect the levels of proliferation. The values at 450 nm (OD450) were measured. Data are expressed as mean ± SD (n=3). *p* value less than 0.05 was considered as significant difference. *p <0.05. ns means no statistical differences.

As shown in **Figure 4**, at two weeks following the third immunization, levels of peripheral T lymphocytes proliferation in the five groups immunized with different combination of protein-delivering *E. faecalis* were all highly significantly higher than that in PBS group and vector control group (*p* < 0.01). Furthermore, the group immunized with combined MDXEF-1/EtAMA1, MDXEF-1/EtIMP1, MDXEF-1/EtMIC2 and MDXEF-1/Et3-1E displayed the highest proliferation levels than other groups with different combinated recombinant *E. faecalis*.

### Mucosal sIgA and sera IgG antibody levels

**Figure 5:**
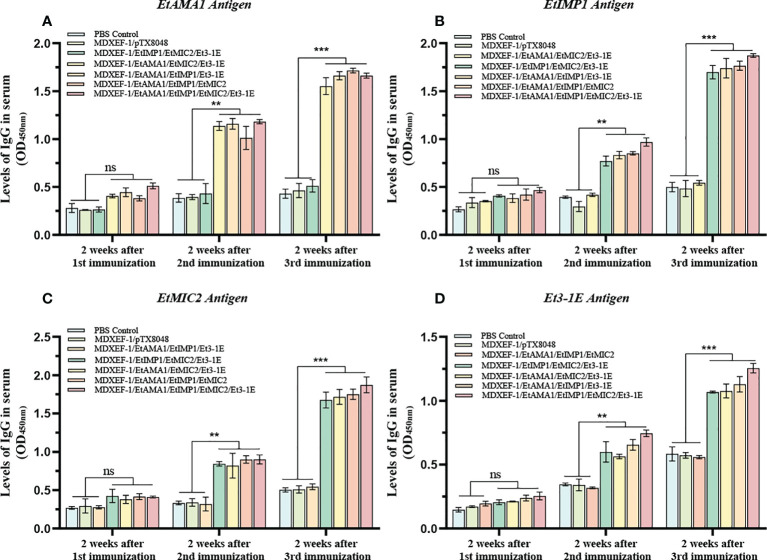
Levels of sera IgG. On day 14 post each immunization, antigen-specific IgG levels in sera was determined by indirect enzyme-linked immunosorbent assay (ELISA). 100 μL (10 μg/ml) of recombinant protein EtAMA1 **(A)**, EtIMP1 **(B)**, EtMIC2 **(C)** and Et3-1E **(D)** expressed in *E coli* BL21 was coated in each well, respectively. Values represent mean ± SD (n=3). **p < 0.01, ***p < 0.001. ns means no statistical differences.

**Figure 6:**
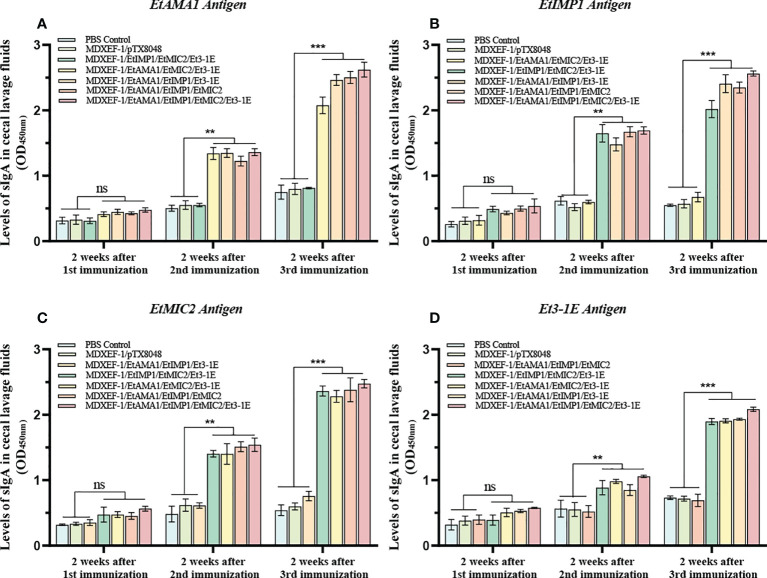
Levels of sIgA in cecal lavage. At two weeks after each immunization, antigen-specific sIgA levels in cecal lavage fluid was determined by ELISA. 100 μL of recombinant EtAMA1 **(A)**, EtMIC2 **(B)**, EtIMP1 **(C)** and Et3-1E **(D)** protein (10 μg/ml) expressed in *E coli* BL21 were coated in each well, respectively. The polyclonal antibody against EtAMA1, EtMIC2, EtIMP1 and Et3-1E protein was used as secondary antibody, respectively. Values represent mean ± SD (n=3). **p < 0.01, ***p < 0.001. ns means no statistical differences.

To clarify the humoral immune state after oral vaccinations, levels of sera IgG and mucosal sIgA were detected and shown in **Figures 5** and **6**. At two weeks after the first round immunizations, levels of sera IgG and sIgA in cecal lavage from chickens (n=3) in each group gradually increased. The significantly higher levels of antigen-specific IgG against EtAMA1, EtIMP1, EtMIC2 and Et3-1E proteins was observed in the five immunized groups compared to PBS control and vector control group (*p* < 0.05) (**Figure 5**). The mucosal sIgA levels in cecal lavage fluid (**Figure 6**) displayed similar changes to IgG levels (*p* < 0.01). The above results indicated that immunizations with three or four combined live recombinant *E. faecalis* expressing different antigens evoked an effective humoral immune responses.

### Levels of cytokines in spleen

**Figure 7:**
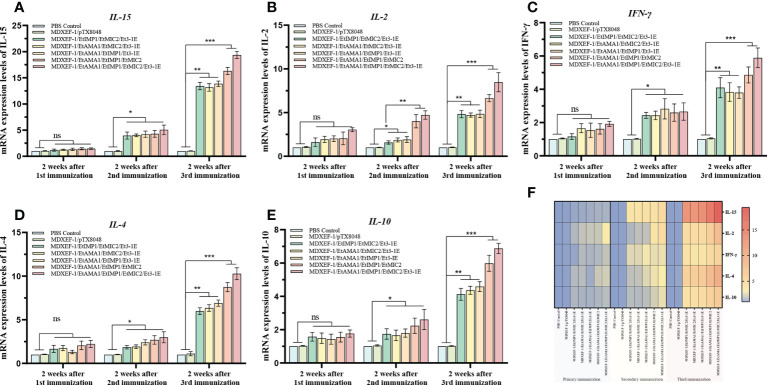
mRNA levels of cytokines in spleen tissue. On day 14 after each immunization, mRNA levels of IL-15 **(A)**, IL-2 **(B)**, IFN-γ **(C)**, IL-4 **(D)** and IL-10 **(E)** in spleen tissues of chickens (n=3) in each group was quantified by qRT-PCR. β-actin was used as reference gene. Heat map of the changing trend of each cytokines after each immunization **(F)**. Data are expressed as mean ± SD (n=3). *p < 0.05, **p < 0.01, ***p < 0.001. ns means no statistical differences.

Immune-related cytokines have immunomodulatory functions, and the levels of key cytokines can reflect the body’s immune state. As shown in **Figure 7**, after the first immunization, statistical differences in mRNA levels of splenic cytokines including IFN-15, IL-γ, IL-4, and IL-10 were not observed among all the groups (*p* > 0.05), while IL-2 displayed a significant increase in the five immunized groups with protein-expressing *E. faecalis* compared to vector control and PBS control group (*p* < 0.05). The levels of IL-15, IL-2, IFN-γ, IL-10 and IL-4 in the combined immunizing groups with five recombinant protein-expressing *E. faecalis* were significantly higher than vector control and PBS control groups (*p* < 0.05) after the second round immunization, and were much considerably higher than the two control groups (*p* < 0.01) after the third immunization. The group immunized with four live recombinant *E. faecalis* delivering EtAMA1, EtIMP1, EtMIC2 and Et3-1E showed the highest levels of cytokines among all the groups. The combined groups with recombinant *E. faecalis* expressing surface-anchored EtAMA1, EtIMP1, EtMIC2 displayed the second higher than other combined groups, including EtAMA1, EtMIC2 and Et3-1E, EtAMA1, EtIMP1 and Et3-1E, and EtIMP1, EtMIC2 and Et3-1E, respectively (*p* < 0.05). There was no significant difference in the levels of these cytokines between the vector control and PBS control group (*p* > 0.05).

### Levels of pro-inflammatory cytokines in ceca

**Figure 8:**
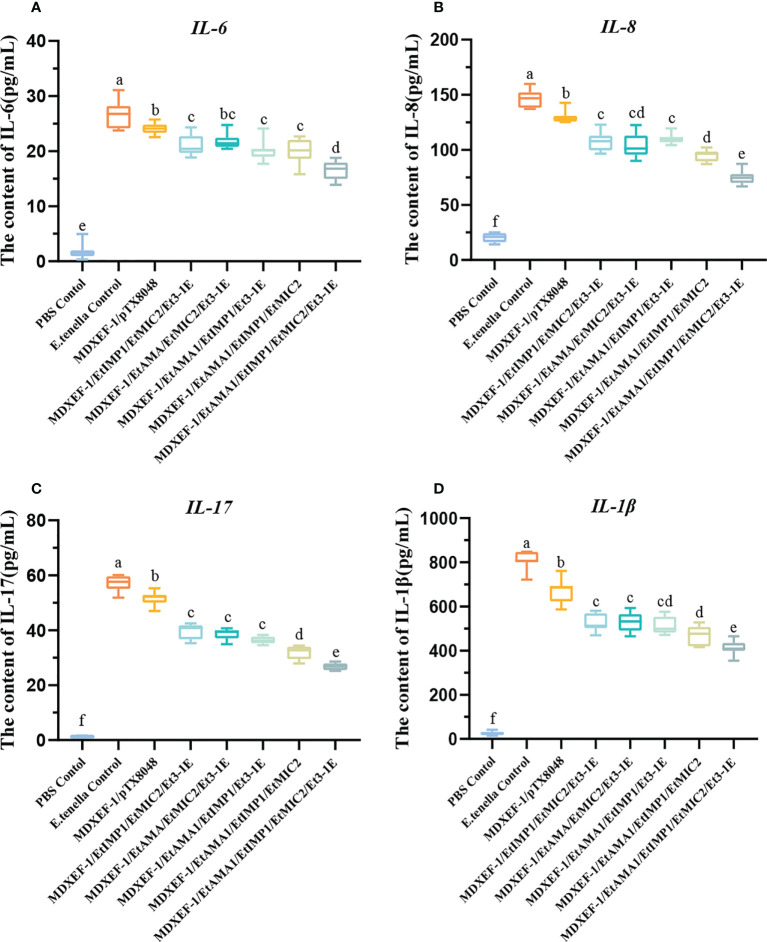
Protein levels of inflammatory cytokines in ceca after *E. tenella* challenge. On day 7 following challenging by *E.tenella*, the content of pro-inflammatory factors including IL-6 **(A)**, IL-8 **(B)**, IL-17 **(C)**, and IL-1β **(D)** in ceca tissues were detected by ELISA. Data are expressed as mean ± SD (n=3). Different small letters showed significantly different (*p* < 0.05).

To demonstrate the levels of pro-inflammatory cytokines in ceca tissues, IL-1β, IL-6, IL-8, and IL-17 were quantified and displayed in **Figure 8**. Compared with *E. tenella*-challenged control group and vector control group, the levels of pro-inflammatory cytokines in other five groups immunized with different combinated protein-expressing *E. faecalis* decreased dramatically (*p* < 0.05). Of note, the group immunized with four combined *E. faecalis* expressing EtAMA1, EtIMP1, EtMIC2 and Et3-1E displayed the lowest levels of IL-1β, IL-6, IL-8, and IL-17 among all the groups (*p* < 0.05).

### Protective efficacy against homologous challenge

**Figure 9:**
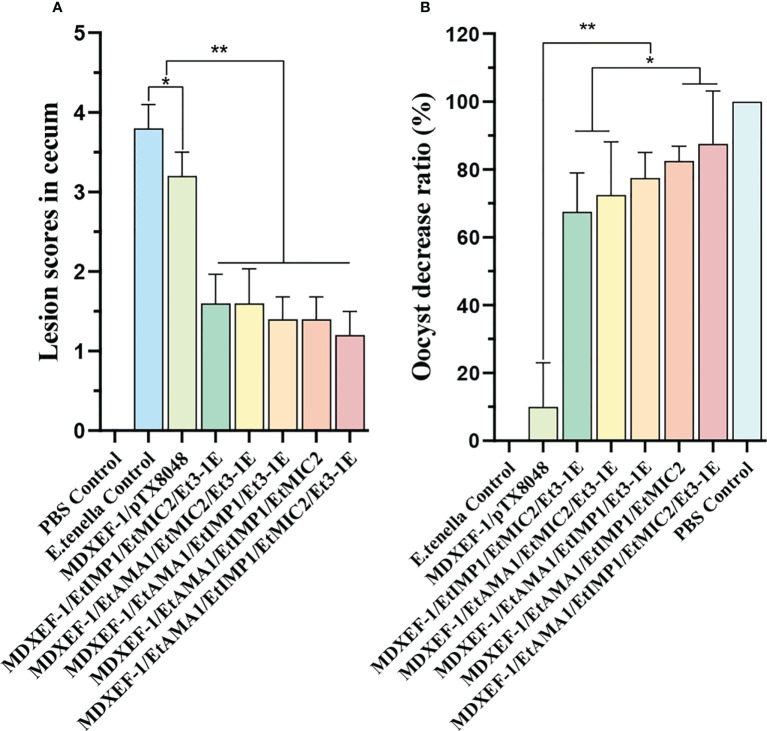
Lesion score in cecum, and oocyst decrease ratio. Lesion scores in cecum were determined at days 7 after *E. tenella* challenge **(A)**. Oocysts output decrease ratio in each group was shown in figure **(B)** Data are expressed as mean ± SD (n=3). *p < 0.05, **p < 0.01.

To demonstrate the protective efficacy, body weight gain of chickens in each group was calculated, and were displayed in **Table 5**. At 59 days of age (on day 7 post challenge), chickens in the five groups orally immunized with three or four combinated protein-delivering *E. faecalis* displayed higher body weight and body weight gain than challenge control and vector control group (*p* < 0.05). The group with four combinated recombinant *E. faecalis*, expressing EtAMA1, EtIMP1, EtMIC2 and Et3-1E showed the highest body weight gain. Compared with *E. tenella* challenge control group and vector control group, the five groups with different combinated protein-delivering *E. faecalis* exhibited lower cecal lesion scores and oocysts output following *E. tenella* infection (*p* < 0.05), and the lowest level of cecal lesion scores and oocysts output were observed in the group with four combined *E. faecalis* delivering surface-anchored EtAMA1, EtIMP1, EtMIC2 and Et3-1E **(** **Figure 9** **)**.

**Table 5:** Averge body weight and body weight gain of chickens in each group.

Groups	Average body weight (g/bird)					Average body weight gain(g/bird)
	10-day-old	24-day-old	38-day-old	52-day-old	59-day-old	
**PBS control**	60.08 ± 7.63	148.53 ± 9.44	263.48 ± 25.59^bc^	433.33 ± 31.85^b^	541.99 ± 52.28^bc^	108.67 ± 34.91^a^
**MDXEF-1/EtAMA1/EtIMP1/EtMIC2/Et3-1E**	63.84 ± 6.00	156.66 ± 14.55	301.36 ± 33.30^a^	498.33 ± 24.29^a^	596.44 ± 25.58^a^	98.11 ± 12.30^a^
**MDXEF-1/EtAMA1/EtIMP1/EtMIC2**	60.91 ± 5.14	152.54 ± 13.61	299.44 ± 35.11^a^	480.77 ± 34.24^a^	571.78 ± 24.53^ab^	91.00 ± 20.93^ab^
**MDXEF-1/EtAMA1/EtIMP1/Et3-1E**	63.19 ± 6.46	153.31 ± 14.48	286.13 ± 31.52^abc^	479.88 ± 26.12^a^	568.33 ± 32.13^ab^	88.45 ± 20.23^ab^
**MDXEF-1/EtAMA1/EtMIC2/Et3-1E**	63.17 ± 6.42	155.32 ± 14.99	297.91 ± 26.22^a^	473.11 ± 54.93^a^	560.89 ± 55.76^abc^	87.77 ± 28.77^ab^
**MDXEF-1/EtIMP1/EtMIC2/Et3-1E**	64.95 ± 5.63	158.15 ± 17.64	287.60 ± 22.57^ab^	480.11 ± 50.73^a^	566.67 ± 52.04^abc^	86.56 ± 30.70^ab^
**MDXEF-1/pTX8048**	60.08 ± 6.39	150.45 ± 9.36	285.19 ± 27.52^abc^	461.11 ± 46.76^ab^	525.44 ± 61.43^c^	61.32 ± 38.95^bc^
***E. tenella* control**	60.65 ± 7.63	147.66 ± 9.44	261.42 ± 30.04^c^	429.23 ± 17.47^b^	481.78 ± 14.17^d^	52.56 ± 13.20^c^

Different small letters means statistical significance (p < 0.05). Body weight gains (BWG) were calculated as follows: BWG = body weight at 7 days post challenge (59-day-old)−body weight when challenging (52-day-old).

### Pathological observation of cecum

**Figure 10:**
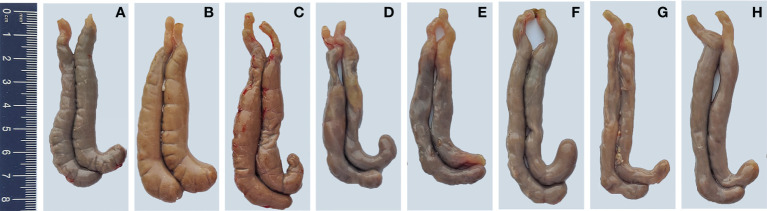
Gross pathological lesions in ceca of chickens. On day 7 post-infection, the gross pathological changes in ceca from infected control group **(B)** and MDXEF-1/pTX8048 group **(C)** showed enlargement and intestinal wall thicken. The ceca of chickens in PBS control group **(A)** and the group with four combined *E. faecalis* MDXEF-1/EtAMA1, MDXEF-1/EtIMP1, MDXEF-1/EtMIC2 and MDXEF-1/Et3-1E, which named MDXEF-1/EtAMA1/EtIMP1/EtMIC2/Et3-1E **(H)** showed no obvious pathological lesions. Other groups with three combined *E. faecalis*, MDXEF-1/EtAMA1/EtIMP1/EtMIC2 **(G)**, MDXEF-1/EtAMA1/EtIMP1/Et3-1E **(F)**, MDXEF-1/EtAMA1/EtMIC2/Et3-1E **(E)** and MDXEF-1/EtIMP1/EtMIC2/Et3-1E **(D)** displayed relatively slight gross pathological changes.

**Figure 11:**
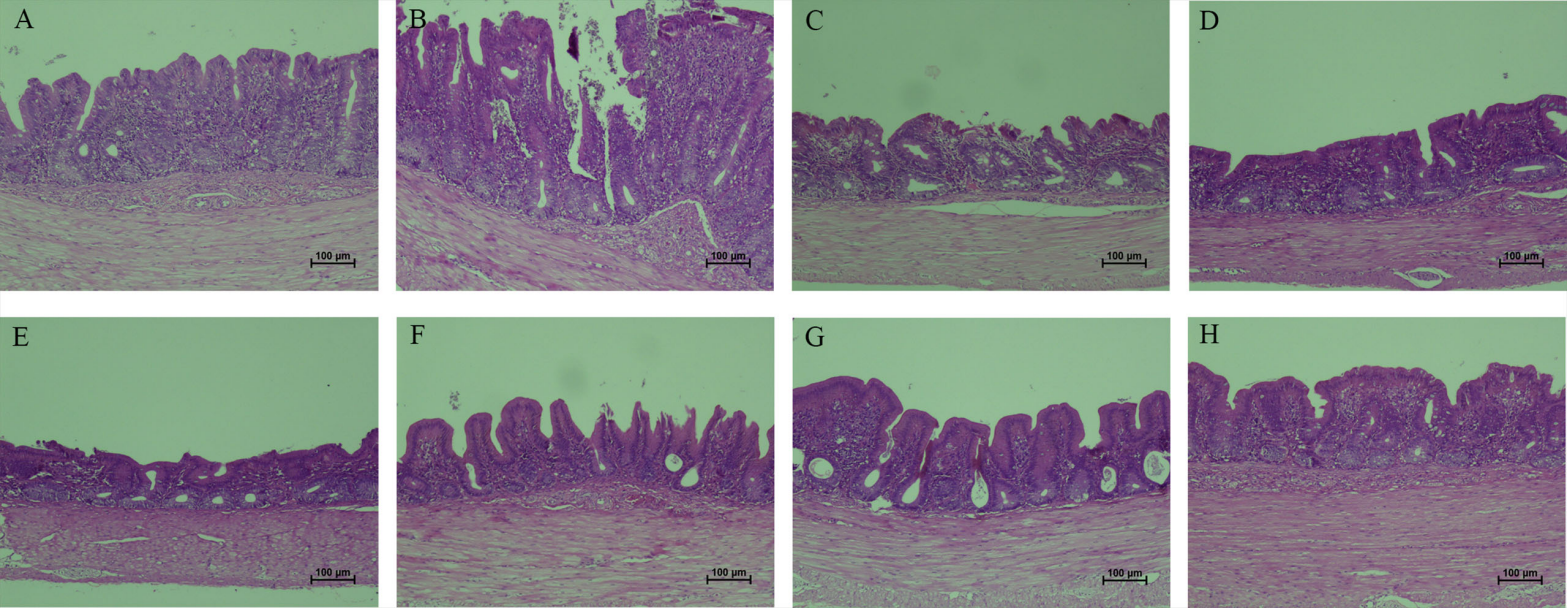
Histopathological changes in ceca tissues from chickens. The typical histopathological changes in ceca were observed in *E. tenella* challenge control **(B)** and MDXEF-1/pTX8048 group **(C)**, showed by severe disrupture of intestinal villi and intestinal glands in lamina propria, and infiltration of scattered inflammatory cells. PBS group **(A)** and the group with four combined *E. faecalis* named by MDXEF-1/EtAMA1/EtIMP1/EtMIC2/Et3-1E **(H)** showed no obvious histopathological changes. The group MDXEF-1/EtIMP1/EtMIC2/Et3-1E **(D)**, MDXEF-1/EtAMA1/EtMIC2/Et3-1E **(E)**, MDXEF-1/EtAMA1/EtIMP1/Et3-1E **(F)**, and MDXEF-1/EtAMA1/EtIMP1/EtMIC2 **(G)** were not severe as *E. tenella* infected group.

To demonstrate the protective effects from the view of pathology, the gross and histopathological changes in ceca were recorded. Ceca of chickens in PBS control group did not show visible gross changes (**Figure 10A**). At 7 days after infection, ceca of chickens in *E. tenella*-infected group (**Figure 10B**) and vector control group (**Figure 10C**) displayed obvious swelling and thickening, while ceca of chickens in the five immunized groups with combined protein-expressing *E. faecalis* displayed relatively slight or no gross pathological changes **Figures 10D–H**. The histopathological alterations were assessed and showed in **Figure 11**. The results revealed that the histopathological lesions in ceca in *E. tenella* challenge control group **(** **Figure 11B** **)** and vector control group **(** **Figure 11C** **)** were severe, including rupture of intestinal villi, hemorrhage, infiltration of inflammatory cells, disintegrated and fragmented intestinal epithelial cells that fall off into the intestinal lumen. Both PBS control group (**Figure 11A**) and the group immunized with four combined protein-expressing *E. faecalis* showed no visible histopathological lesions (**Figure 11H**). The histopathological changes in the other four immunized groups were relatively mild (**Figures 11D–G**), showing the less damaged intestinal villi and structurally intact epithelial cells, and infiltrated inflammatory cells.

## Discussion

Avian coccidiosis causes severe economic losses to the poultry industry worldwide. The large-scale application of anticoccidial chemical drugs and long-term used live attenuated parasites vaccines has resulted in substantial drug resistance and reversion virulence (30). Nowadays, exploration of novel vaccines against chicken coccidiosis has emerged to be hot research topics. Considering a variety of proteins are involved during invasion of *E. tenella* into host cells, in the present study, four key proteins that related to immune responses or invasion, including *E. tenella* AMA1 (EtAMA1), *E. tenella* IMP1 (EtIMP1), *E. tenella* MIC2 (EtMIC2) and *E. tenella* 3-1E (Et3-1E) were chosen to develop recombinant live oral vaccine. EtAMA1 is type I transmembrane protein secreted on the surface of *E. tenella* sporozoites during invasion, and the expression levels of EtAMA1 at sporozoite stage is higher than other stages, and therefore is crucial for sporozoites invasion (31). Previous studies have proved that EtAMA1 protein also evoked immune responses and offered partial protection against *E. tenella* challenge (18, 25). EtIMP1 was reported to be a candidate protective antigen, and chickens immunized with recombinant EtIMP1 protein delivered by *Lactococcus lactis* (26) or *E. coli* (32) elicited effective immune responses and provided partial resistance to *E. tenella* infection. Et3-1E protein plays an important role during invasion of *E. tenella*, and induces immune responses. Et3-1E protein expressed in *Bacillus subtilis* induced protective immunity against *E. tenella* challenge (12). EtMIC2 is secreted by microneme, and its complex with other proteins locates on the host-parasite interface during attachment, and then translocates to the posterior end of parasites during penetration of host cells (33). It was reported that immunization with recombinant EtMIC2 protein were found to reduce oocysts output from infected chickens (34).

In the present study, the above four proteins were selected to prepare oral vaccine to induce specific immune responses to kill parasites or inhibit parasites binding and the subsequent invasion into host cells. When we plan to explore novel tools to deliver the selected four target proteins, lactic acid bacteria (LAB) interested us. LAB are recently reported to be a vehicle to deliver promising candidates to stimulate effective intestinal mucosal and systemic immune responses (35, 36). Our previous experiments showed that probiotic *E. faecalis* displaying surface-anchored *E. tenella* 3-1E protein (14), and Hexon protein of fowl adenovirus 4 (FAdV-4) (22) offered partial immune protective efficacies against homologous infection. Therefore, probiotic *E. faecalis* was used as delivery tools to express target proteins. It has been reported surface-anchoring proteins based on cell wall anchoring (CWA) motif evoked more effective specific immune responses than cytoplasmic or secreted protein (13, 25). In addition, dendritic cell targeting peptide (DCpep) consisting of twelve amino acids was recorded to specifically targeting to dendritic cells (37) and antigen-specific immune responses were enhanced by fusing with DCpep (38). DCs are the professional antigen presenting cells, and play a vital role in fighting viral infections and other diseases (39, 40). Considering fusion of four key proteins together is complicated, four recombinant *E. faecalis* that delivering single surface-anchored objective protein fusing with DCpep were prepared, respectively.

Nowadays, both cellular and humoral immunity are generally accepted to play vital role on resistance of *Eimeria* infection. In the current study, significant levels of antigen-specific sera IgG and sIgA in cecal lavage fluid were observed from all the five groups immunized with combined protein-delivering *E. faecalis*, indicating oral immunization with combined recombinant *E. faecalis* triggered specific intestinal mucosal and systemic humoral immune responses. Intestinal mucosal immunity is an important line of defense against pathogens that transmitted *via* oral route, and it was reported that sIgA produced in local mucosa sites competitively binds to *Eimeria* parasites, which inhibits the subsequent parasites invasion into host cells (41). The levels of antibodies against each individual antigen were separately tested with the aims to detect the cross-reactivity among all the four target antigens. The levels of antigen-specific IgG in sera and sIgA in cecal lavage fluid in the groups immunized with any three combinated protein-delivering *E. faecalis* did not show statistical differences with PBS group when the fourth antigen that was not contained in the three combination was coated in the plates, which suggesting that there was no cross reaction among the four protein antigens.

The antigen-specific cellular immune responses stimulated by each recombinant *E. faecalis* were investigated. Cytokines are a group of proteins that synthesized and secreted by immune cells and non-immune cells upon stimulation, which exert important biological functions. CD4^+^ T cells are classified into Th1 and Th2 types, which secret IFN-γ, IL-2 and IL-15 that related to cellular immune responses, and IL-4, IL-10 that regulate production of antibodies (42). In the present study, the levels of cytokines IFN-γ, IL-2, IL-15, IL-10 and IL-4 in spleen tissues of experimental chickens from the five groups immunized with combined protein-delivering *E. faecalis* were significantly upregulated compared to vector control (MDXEF-1/pTX8048) and PBS control group (*p* < 0.05), indicating that oral vaccination with recombinant antigen-expressing *E. faecalis* effectively activate Th1 and Th2 type cellular immune responses in chickens. Notably, the group immunized with four combined recombinant *E. faecalis* MDXEF-1/EtAMA1/EtIMP1/EtMIC2/Et3-1E showed the highest levels in both cellular and humoral immunity. The above results suggested that combinated application of multi-antigenic protein is a promising method to design *Eimeria* recombinant subunit vaccines.

To evaluate the immuno-protective efficacy against homologous challenge, in this work, vaccinated chickens were then subjected to infection with *E. tenella.* The relative body weight gain, oocyst reduction ratio, cecal lesion scores and histopathological changes were assessed. The results showed that groups with three or four combination of protein-expressing *E. faecalis*, especially the four combinated group, displayed significantly reduced oocyst shedding, alleviated cecal damage and decreased average lesion scores in ceca compared to chickens in vector control group. The combination of four recombinant *E. faecalis* showed the best protective effects in this study, which are similar to other reported work (43), suggesting that combination of multi-antigen stimulated more effective cellular immune responses as well as humoral and intestinal mucosal immune responses, and therefore provided more effective protective effects against *Eimeria* damage to gut tissues.

*Eimeria* infection activated obvious intestinal inflammatory responses, which play a critical role in resisting parasite challenge (44). However, the exaggerate inflammatory responses also cause severe inflammatory injury to intestinal epithelial cells, which reduced absorption of nutrients in intestine. To reflect inflammatory responses in cecal epithelial cells, several pro-inflammatory factors in cecal tissues were detected. Compared to challenge control and vector control group, the groups immunizing with combined protein-expressing *E. faecalis*, especially the group with combined EtAMA1-, EtIMP1-, EtMIC2- and 3-1E-expressing *E. faecalis* displayed dramatically decreased levels of inflammatory factors (*p* < 0.01). The above results suggested that the enhanced immune responses reduced the parasites load and stimuli in ceca, and therefore alleviated intestinal inflammatory injury.

Considering the immune protective effects provided by oral administration of combined recombinant *E. faecalis* delivering important antigens were far lower than that offered by live vaccines consists of several attenuated *Eimeria* species, in the present study the groups with live vaccines were not designed. However, this novel platform still showed more attractive and promising potential application in future, mainly for its safety, convenient preparation and immunization, and also the stimulated effective immune responses. This platform could be applied not only in the field of *Eimeria* research but also in other avian disease. To our knowledge, this is the first report using combination of probiotics *E. faealis* delivering different antigen to activate immune responses against *E. tenella*.

## Conclusions

In the present study, four recombinant probiotics *E. faecalis* that delivering surface-anchored dendritic cells target peptide (DCpep) fused with key *E. tenella* proteins EtAMA1, EtIMP1, EtMIC2, and Et3-1E were constructed, respectively. The animal immunization test revealed that chickens orally administrated with three or four, especially the four combined recombinant *E. faecalis* stimulated specific intestinal mucosal, cellular and humoral immune responses, which provided anti-coccidial effects displaying by the alleviated cecal injury and reduced weight loss. This study provide a meaningful reference for exploration of novel vaccines against coccidiosis.

## Data availability statement

The raw data supporting the conclusions of this article will be made available by the authors, without undue reservation.

## Ethics statement

The animal study was reviewed and approved by ethics committee for animal sciences regulations at Northeast Agricultural University, Heilongjiang Province, China.

## Author contributions

DM and CM designed the study. WZ, HC, BB, ZJ, XP, BW, RK, and QL prepared the experimental materials. CM and HC contributed to the analytic tools. WZ, HC, and CM analyzed the data. WZ and HC wrote the paper. CM and DM revised the manuscript. All authors contributed to the article and approved the submitted version.

## Funding

This study is funded by grants from National Natural Science Foundation of China (31973003).

## Conflict of interest

The authors declare that the research was conducted in the absence of any commercial or financial relationships that could be construed as a potential conflict of interest.

## Publisher’s note

All claims expressed in this article are solely those of the authors and do not necessarily represent those of their affiliated organizations, or those of the publisher, the editors and the reviewers. Any product that may be evaluated in this article, or claim that may be made by its manufacturer, is not guaranteed or endorsed by the publisher.
