# Supplementary material for: Combined oral immunization with probiotics Entercoccus faecalis delivering surface-anchored Eimeria tenella proteins provide protective efficacies against homologous infection in chickens
Source: Front Immunol. 2022 Oct 13;13:1042143. doi: 10.3389/fimmu.2022.1042143 (PMC9606674; doi:10.3389/fimmu.2022.1042143)
Supplement: Supplementary file 1 [file DataSheet_1.docx]

Supplementary Table

# Supplementary Tables

Table S1 Accession number of gene sequences from NCBI GeneBank

| Gene name | Accessin NO. | Link in GeneBank |
| --- | --- | --- |
| EtAMA1 | JN032081.1 | https://www.ncbi.nlm.nih.gov/nuccore/JN032081.1 |
| EtIMP1 | KC215109.1 | https://www.ncbi.nlm.nih.gov/nuccore/452818071 |
| EtMIC2 | KC333870.1 | https://www.ncbi.nlm.nih.gov/nuccore/KC333870.1 |
| Et3-1E | EF426471.1 | https://www.ncbi.nlm.nih.gov/nuccore/EF426471.1 |
| β-actin | NM_205518.2 | https://www.ncbi.nlm.nih.gov/nuccore/NM_205518.2 |
| IL-2 | NM_204153.2 | https://www.ncbi.nlm.nih.gov/nuccore/NM_204153.2 |
| IL-4 | NM_001007079.2 | https://www.ncbi.nlm.nih.gov/nuccore/NM_001007079.2 |
| IL-10 | NM_001004414.4 | https://www.ncbi.nlm.nih.gov/nuccore/NM_001004414.4 |
| IL-15 | NM_204571.2 | https://www.ncbi.nlm.nih.gov/nuccore/NM_204571.2 |
| IFN-γ | NM_205149.2 | https://www.ncbi.nlm.nih.gov/nuccore/NM_205149.2 |
